# The Effect of Bio-Electromagnetic Energy Regulation Therapy on Erectile Dysfunction in Patients with Multiple Sclerosis: A Triple-Blind Randomized Clinical Trial

**DOI:** 10.3390/jcm13237060

**Published:** 2024-11-22

**Authors:** Abdulaziz Ali Y. Alzharani, Ali M. Alshami, Turki Abualait, Hatem Al Azman, Foziah Jabbar Alshamrani, Yahya Hilal Alzahrani, Youssef A. Althobaiti

**Affiliations:** 1 College of Applied Medical Sciences, Imam Abdulrahman Bin Faisal University, Dammam 31451, Saudi Arabia; 2210700007@iau.edu.sa (A.A.Y.A.);; 2 Department of Physical Therapy, Armed Forces Center for Health Rehabilitation, Taif 26526, Saudi Arabia; 3 Department of Neurology, King Fahd Hospital of the University, Al Khobar 334445, Saudi Arabia; 4 Department of Neurology, King Abdulaziz Specialist Hospital, Taif 26521, Saudi Arabia

**Keywords:** multiple sclerosis, BEMER, sexual dysfunction, erectile dysfunction

## Abstract

**Objectives**: To evaluate the effect of bio-electromagnetic energy regulation (BEMER) therapy on erectile dysfunction (ED) in patients with multiple sclerosis (MS). **Methods:** A triple-blind randomized clinical trial was conducted in two different centers. Fifty-two male participants with MS were randomly allocated into two groups. Patients received either three weeks of BEMER with pelvic floor exercises or sham BEMER with pelvic floor exercises. The primary measure was the International Index of Erectile Function—Erectile Function (IIEF-EF). Secondary measures included the Sexual Health Inventory for Men (SHIM), Erection Hardness Scale (EHS), Arizona Sexual Experience Scale (AXES), Modified Fatigue Impact Scale (MFIS), and Multiple Sclerosis, Intimacy, and Sexuality Questionnaire (MSISQ-19). **Results**: Compared to the sham BEMER group, the BEMER group showed better improvements in the IIEF-EF (mean difference [MD]: −6.9, *p* < 0.001), SHIM (MD: −6.1, *p* < 0.001), EHS (MD: −0.4, *p* = 0.022), AXES (MD: 2.9, *p* = 0.030), MSISQ-19 (MD: 15.0, *p* < 0.001), and MFIS (MD: 31.0, *p* < 0.001). **Conclusions**: BEMER therapy improved erectile function and sexual satisfaction and reduced fatigue in patients with MS after three weeks of intervention. Long-term follow-up studies are warranted to ascertain the sustained benefits of BEMER therapy for MS-related ED.

## 1. Introduction

Multiple sclerosis (MS) is an autoimmune disease that affects the central nervous system (CNS) and often leads to physical, cognitive, and neurological problems in young adults [1]. The global prevalence of MS is around 35.9 cases per 100,000 people, with approximately 2.8 million cases worldwide [2]. Approximately 309 cases of MS are reported per 100,000 people in the United States [2]. In Europe, the prevalence ranges from 100 to 200 cases per 100,000, with some Northern European countries reporting up to 300 per 100,000 [2]. People with MS report a wide range of symptoms, including fatigue, weakness, spasticity, gait and balance problems, cognitive and visual impairment, bladder and bowel dysfunction, sensory loss, and neuropathic pain [3,4].

Sexual dysfunction (SD) is also a common complication [5], affecting 40 to 80% of females and 50 to 90% of males [5]. SD among males with MS includes erectile dysfunction (ED) (50–75%), ejaculation disorder (50%), decreased libido (39%), and anorgasmia (37%) [6,7]. ED is prevalent among MS patients, occurring in over 62% of cases [8,9]. ED is the inability to achieve and maintain an erection that allows for satisfactory sexual performance [10]. Medications, vacuum constriction devices, and penile prostheses are common treatment options for ED [6,11]. Physical therapy has also been prescribed to patients with ED [11]. It includes interventions such as light-to-moderate aerobic exercise and pelvic floor muscle treatments such as therapeutic exercise, biofeedback, and electrical stimulation [12,13].

Therapeutic interventions utilizing electromagnetic fields, such as bio-electromagnetic energy regulation (BEMER), have emerged as promising tools in physical therapy [14,15,16]. BEMER therapy is a non-invasive technique that improves tissue microcirculation by activating deeper anatomical structures without discomfort [16,17]. Few studies have examined the effect of magnetic therapy on erection in animals [18] and individuals with neuropathic [15] and psychogenic ED [14], but all studies have demonstrated positive findings.

To our knowledge, there is a lack of studies on the effect of magnetic therapy, particularly BEMER, on ED in patients with MS. Therefore, the aim of this study was to evaluate the impact of BEMER on ED in such patients. We hypothesize that a three-week intervention combining daily 20-min BEMER therapy sessions with pelvic floor muscle training will significantly enhance erectile function in patients with MS. BEMER therapy improves microcirculation [19,20,21], which is essential for proper penile blood flow. Pelvic floor exercises strengthen the muscles responsible for penile rigidity, as highlighted by Prota et al. [22] and Dorey [23]. The three-week duration of this intervention is supported by studies which show the short-term efficacy of BEMER in improving circulation [16,17,24,25,26,27]. By targeting both vascular and muscular factors, this dual-pronged approach presents a promising strategy for improving sexual function in MS patients.

## 2. Materials and Methods

This triple-blind randomized clinical trial was conducted at the neurology departments of two hospitals. The patients and assessors were blinded to the type of treatment, and the treating therapist was also blinded to the results of the outcome measurements. The study followed the Declaration of Helsinki and complied with Consolidated Standards of Reporting Trials (CONSORT) guidelines [28]. The study was prospectively registered at ClinicalTrials.gov (NCT05615454, date: 11 June 2022) and conducted between January and December 2023. Ethical approval was obtained from two institutional review boards. All participants provided written consent before participating in the study.

## 3. Sample Size Calculations

The sample size was calculated using statistical software (G*Power 3.1.9.7, the Heinrich-Heine-University, Düsseldorf, Germany) based on data obtained from a previous study [29]. The following combination was used to calculate the sample size: the analysis of covariance (ANCOVA), fixed effect, main effect, interactions, an ES of 0.87, an alpha level of 0.05, a power (1-β) of 95%, a numerator df of 10, two groups, two measurements (time points), and one covariate. After accounting for a 25% attrition rate, we recruited a total of 52 participants, with 26 in each group.

## 4. Participants

Men diagnosed with relapsing–remitting MS according to the revised McDonald criteria [30] were included in the study if they were 18–40 years old, had a score of 6 and 21 on the International Index of Erectile Function 5 (IIEF-5), scored ≤ 6 on the Expanded Disability Status Scale (EDSS) [31], and had engaged in sexual activity in the past month. Notably, IIEF-5 is a tool for quickly assessing erectile function through five questions scored from 0 to 4, leading to total scores ranging from 0 to 25. Scores of 0–7 indicate severe erectile dysfunction, 8–11 indicate moderate dysfunction, 12–16 reflect mild-to-moderate dysfunction, 17–21 indicate mild dysfunction, and 22–25 signify no dysfunction [32]. The IIEF-5 is widely used in clinical practice and research to evaluate treatment effectiveness and guide management strategies.

Participants were excluded if they had experienced clinical relapse within one month before the experimental study date, had a cognitive impairment that affected their ability to answer the questionnaires, had a history of chronic illness such as epilepsy or diabetes, had an acute bacterial infection causing fever, had received medication or treatment for ED in the past 7 days, had implanted metal devices or pacemakers, or had previously undergone treatment with a pulsed electromagnetic field.

## 5. Randomization and Blinding

An independent person who was not involved in the study created a randomization sequence with an allocation ratio of 1:1. Two independent assessors—neurologists—assessed the participants’ eligibility, collected primary demographic data, and performed all the outcome measurements before and after treatment. Eligible participants picked a sequentially numbered concealed opaque envelope (SNOSE) [33] from a box and presented it to the treating therapist (the only person who knew the group assignment). We stored the data in a locked box, which the therapist did not access until the end of the trial.

## 6. Intervention

A total of 15 sessions were delivered (5 sessions per week over 3 weeks). The first session lasted an hour and involved collecting sociodemographic data and outcome measurements before administering the treatment.

**Figure 1:**
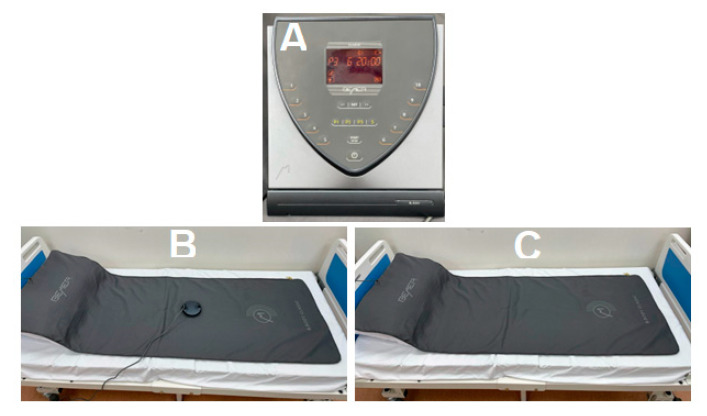
(**A**) BEMER control unit, (**B**) B. Spot. (**C**) Full-body mattress.

The participants in the BEMER group were instructed to lie supine on a full-body mat connected to a BEMER device (model type: B.BOX CLASSIC, model NO: 420100, 12–15 Volt from BEMER Int. AG, Liechtenstein), which was placed over the treatment bed (Figure 1). The B. Spot (an application module focused on therapy for specific body regions) was switched on and placed over the genital area without removing clothes. Then, the BEMER mat was activated, and the intensity setting was increased using program 3, which was 20 min long and included intensity levels of 6–10 units. The three-week duration of our intervention aligns with studies demonstrating that short-term BEMER therapy effectively enhances circulation and leads to measurable physiological improvements [24,25,26,27,28,29]. This evidence might reinforce the feasibility of a three-week intervention. Additionally, these settings were chosen according to the manufacturer’s recommendations, where the exposure therapy session may last 20 min daily for three weeks.

The BEMER parameters were calibrated and tested before the sessions. The participants in the sham BEMER group underwent the same treatment as those in the BEMER group except that the B. Spot and mat were deactivated. After BEMER, both groups performed pelvic floor exercises during the sessions.

For pelvic floor muscle exercises, the therapist asked the participants to tighten their pelvic floor muscles (ischiocavernosus and bulbocavernosus muscles) as much as possible (as if to prevent flatus from escaping) to achieve muscular contraction during the session and at home. The therapist assessed the exercises to ensure that the muscles functioned strongly, by manual palpation, and could retract the penis and elevate the scrotum. The participants were instructed to maximally contract their pelvic floor muscles in the supine, sitting, and standing positions for 10 s and with three repetitions nine times daily in all positions [34,35,36]. The therapist reviewed the exercises with them during their visits to guarantee compliance.

## 7. Outcome Measurements

The assessors measured all the outcomes at baseline before treatment and after the treatment in the last session. The primary outcome measure was the International Index of Erectile Function—Erectile Function (IIEF-EF). The IIEF-EF domain score is a patient questionnaire used to measure different aspects of erectile performance and evaluate disease severity in efficacy trials regarding ED. The questionnaire consists of 6 questions out of the 15 questions in the IIEF-15 questionnaire [37]. The questions included items 1, 2, 3, 4, 5, and 15. The Arabic version of the IIEF-15 questionnaire used in this study had been validated through patient-centered outcome assessment (PCOA).

The secondary outcome measurements included the following: First, the International Index of Erectile Function (IIEF-5), also known as the Sexual Health Inventory for Men (SHIM), was determined. This was utilized both in the eligibility criteria of this study and as a secondary outcome measurement. It consists of a five-item questionnaire that can distinguish patients with ED. It classifies ED into five degrees of severity, ranging from none (22–25) to severe (5–7). This study used the validated Arabic version of the IIEF-5 [38]. Second, the Arizona Sexual Experience (ASEX) scale was designed to assess five significant aspects of SD: drive, arousal, penile erection/vaginal lubrication, capability of achieving orgasm, and orgasmic satisfaction. The items are measured on a 6-point scale (1–6), with higher scores reflecting impaired sexual function. Sexual dysfunction was defined as a total score ≥ 19, any one item with a score of 5, or any three items with a score ≥ 4. The Arabic version of the ASEX is reliable and valid [39] and was used in the current study. Third, the Erection Hardness Scale (EHS) was used to rate the hardness of a penile erection. The EHS comprises a 5-point Likert scale from 0 (the penis does not enlarge) to 5 (the penis is completely hard and fully rigid). The EHS has demonstrated good responsiveness in clinical trials for therapy development [40]. The current study used the validated and reliable EHS Arabic version [41]. Fourth, the Multiple Sclerosis, Intimacy, and Sexuality Questionnaire (MSISQ-19) is a widely used and accurate 19-item tool for exploring the impact of MS on sexual function. It evaluates the effect of MS symptoms on sexual activity and overall sexual satisfaction during the preceding six months. Each item is scored on a Likert scale ranging from 1 to 5. The MSISQ-19 provides scores on three different subscales: primary SD, secondary SD, and tertiary SD. The validated Arabic version of the MSISQ-19 was used in this study [42]. Fifth, the Modified Fatigue Inventory Scale (MFIS) is a comprehensive 21-item questionnaire used to collect information about the impact of physical (9 items), psychosocial (2 items), and cognitive (10 items) fatigue over the past 4 weeks. The MFIS uses a 5-point Likert scale ranging from never (0) to always (4), and 15 points or more are considered to indicate a clinically significant alteration in fatigue in patients with MS. The Arabic version of the MFIS is valid and reliable and was used in this study [43].

## 8. Statistical Analysis

Data were analyzed using the statistical software IBM SPSS version 25 (SPSS, Inc., Chicago, IL, USA). Means and standard deviations are used to represent descriptive statistics for continuous variables. The data were tested for normal distributions using the Shapiro–Wilk test, and all the variables were normal except age.

The two groups’ continuous and categorical variables were compared at baseline using an independent t-test and a chi-squared test, respectively. ANCOVA was performed to compare the outcome measurements between the two groups using the difference in mean change. Levene’s test and normality checks were performed to confirm all the assumptions. Bonferroni corrections were conducted. Partial eta-squared (η^2^) was calculated and used to indicate small effects (0.01), medium effects (0.06), and large effects (0.14) [44]. Differences were considered statistically significant at *p* < 0.05.

## 9. Results

**Figure 2:**
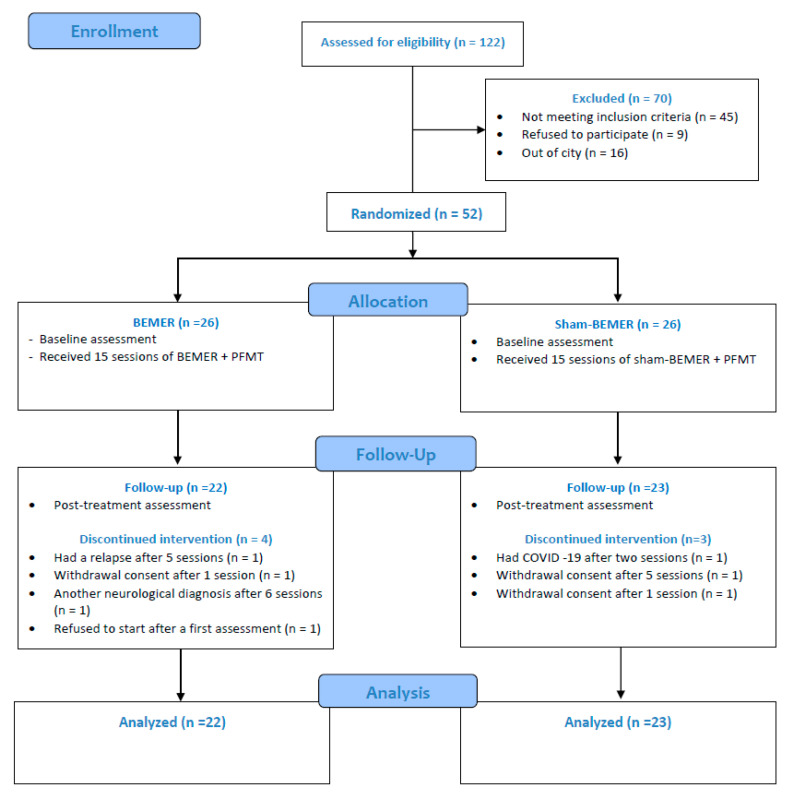
Flowchart of participants throughout randomized clinical trial. PFMT: pelvic floor muscle training.

A total of 122 individuals were considered for enrollment in the study. Of these, 70 participants were excluded for various reasons (Figure 2). The remaining 52 participants met the inclusion criteria and were randomized into one of the groups. Four participants in the BEMER group and three in the sham BEMER group did not complete the follow-up sessions. As a result, the final analysis included 22 participants in the BEMER group and 23 participants in the sham BEMER group.

Table 1 presents the baseline characteristics of the groups. Both groups were similar in all variables except that the BEMER group had a higher body mass index (BMI) (26.6 kg/m^2^) compared to the sham BEMER group (22.1 kg/m^2^). A total of 45 participants were classified according to their level of ED using the SHIM. Out of the 45 participants, only 4 (8.9%) had severe ED, and the majority of participants had mild to moderate ED. Fewer than half of the participants were smokers in each group, but the majority of participants in both groups did not have regular exercise habits.

Based on the MSISQ-19 scoring, 81.3% of the participants in the BEMER group had tertiary SD, while 66.7% of the participants in the sham BEMER group had secondary SD. Almost all the participants reported that their doctors did not ask them about their SD and ED. In both groups, 80% of the participants did not inform their doctors about their ED. Most of the participants preferred a male physician when they wanted to discuss and treat ED.

The ANCOVA revealed improvements in the BEMER group in all the primary and secondary outcome measurements compared with the sham BEMER group with moderate-to-high effect sizes (Table 2). The BEMER group demonstrated better erection function in the IIEF-EF (mean difference [MD] = −6.9, *p* < 0.001, Partial Eta Squared = 0.310) and SHIM (MD = −6.1, *p* < 0.001, Partial Eta Squared = 0.317). The group also demonstrated improvements in erection hardness (EHS: MD = −0.4, *p* < 0.022, Partial Eta Squared = 0.122), enhanced sexual experiences (AXES: MD = 2.9, *p* < 0.030, Partial Eta Squared = 0.110; MSISQ-19: MD = 15.0, *p* < 0.001, Partial Eta Squared = 0.261), and less fatigue (MFIS: MD = 31.0, *p* < 0.001, Partial Eta Squared = 0.456).

Regarding within-group differences, the BEMER group improved significantly in all the outcome measurements after treatment compared to baseline. However, the sham BEMER group improved only in EHS and AXES, but this improvement had only a small effect size. Notably, no participant developed or reported any harmful or unintended effects in either group.

## 10. Discussion

This randomized controlled trial (RCT) investigated the effectiveness of BEMER compared with sham BEMER for treating ED in patients with MS. In comparison to sham BEMER, the BEMER group exhibited significant improvements in IIEF-EF, SHIM, and EHS scores, along with decreases in AXES, MSISQ-19, and MFIS scores. The PEMF and BEMER are similar types of magnetic therapy. Previous research investigated the impact of PEMF therapy on individuals with neuropathic [15] and psychogenic ED [14] and demonstrated comparable improvements in erectile function, which supports the efficacy of BEMER therapy. However, studies on the effect of BEMER on ED in patients with MS have been lacking.

BMI is known as a risk factor for ED [45] and was higher in the BEMER group in this study. While randomization aims to create balanced groups, minor baseline differences, such as those in BMI, can still arise by chance. To account for this potential variability, we included BMI as a covariate in our ANCOVA analysis. Our results remained robust, indicating that these baseline differences did not influence the primary outcomes. Our rigorous randomization process and the lack of evidence for systematic bias further support the validity of our findings. This approach aligns with established practices in clinical trial design and analysis. These participants experienced significant improvements in erectile function [46,47,48]. However, many studies offer valuable insights into how a higher BMI may affect the development and progression of MS [49,50]. Existing evidence suggests that BMI serves not only as a general marker of health risk but may also significantly impact the onset and severity of autoimmune conditions such as MS [49,50,51,52]. This relationship implies that addressing BMI could be essential in preventing and managing MS [53]. The BEMER group showed improvement in all outcomes despite just over a third (36.36%) having a history of smoking, which is a risk factor for ED [54]. Notably, research indicates that quitting smoking may further improve erection and sexual performance [55]. Over half of the BEMER group reported no physical activity or exercise. The literature supports a correlation between exercise and improved erectile function [56]. Encouraging physical activity and exercise can help reduce risk factors for ED in individuals with MS.

The baseline MSISQ-19 results revealed that 11.1% of the participants had primary SD, 53.3% had secondary SD, and 35.6% had tertiary SD. A previous study showed a high prevalence of SD among patients with MS (54.8% with primary, 45.2% with secondary, and 50.3% with tertiary SD), and over 80% of them reported difficulty in achieving an erection [42]. We found that 80% of patients never discussed ED with their physicians. Similarly, only 2.2% of neurologists talked about ED and SD with patients who have MS. This lack of communication could be due to cultural barriers or stigma. Our results are consistent with previous studies that showed low rates of inquiry and disclosure about sexual issues among neurologists and patients with MS. A study in France found that only 30% of neurologists asked their patients about their sexual health, and 63% of patients had never discussed sexual issues with their doctors [57]. Another study in Austria reported that only 15% of neurologists had conversations with their patients about sexuality [58]. This highlights the need for increased attention to the issue of ED in patients with MS.

Our study has shown that 3 weeks of BEMER therapy effectively enhances erectile function. This was evidenced by the marked improvements in the IIEF-EF, SHIM, and EHS scores. Additionally, the patients who received BEMER treatment had enough erectile function to engage in complete sexual intercourse, as evidenced by the IIEF-EF, SHIM, and EHS scores. Moreover, the results demonstrated an improvement in the ASEX score for sexual satisfaction after receiving BEMER therapy.

The current study revealed that the BEMER treatment reduced fatigue symptoms, as demonstrated by the MFIS. This is consistent with previous studies that showed decreased fatigue in patients with MS after BEMER therapy [27,59]. Fatigue symptoms may dominate ED in men with MS and affect their sexual function and satisfaction [60]. Therefore, the BEMER intervention may have dual benefits for patients with MS, as it may improve both fatigue and erectile function.

BEMER therapy may improve ED in patients with MS through several mechanisms. It enhances tissue microcirculation by increasing microvessel vasomotion, arteriovenous pO2 differences, the number of open capillaries, and blood flow rates [61]. These effects have been shown using high-resolution microscopy and laser reflection spectroscopy [62]. Recent studies highlight the effectiveness of BEMER therapy. Research by Gyulai et al. [16] showed significant improvements in microcirculation for chronic low back pain patients. Auger et al. [24] found that combining BEMER therapy with osteopathic treatment reduced pain and improved quality of life. Additionally, Moen et al. [63] reported positive effects on sleep quality among elite female football players, while Kreska et al. [64] noted improved heart rate variability in patients with coronary heart disease. These findings suggest that BEMER therapy provides therapeutic benefits for various health conditions, potentially improving ED in patients with MS.

The exploration of BEMER therapy in the context of treating ED in patients with MS holds promising clinical implications. Integrating BEMER therapy with pelvic floor muscle exercises could offer a non-invasive, safe approach to enhancing treatment efficacy in patients with MS. However, realizing the full potential of this integrated approach requires increased awareness and communication between healthcare professionals and these patients regarding sexual health.

Creating a supportive and open dialog around sexual health can contribute significantly to improving patient outcomes. Moreover, approaching ED discussions with sensitivity and respect is essential for patient comfort and adherence to treatment recommendations, fostering a trusting patient–provider relationship that is integral to successful therapeutic interventions. A strength of the current study is that it followed a robust study design with a blinded RCT, which has the advantage of reducing bias and ensuring the validity of the results. However, some limitations should be considered. First, the study lacked long-term follow-up, which limits our understanding of the observed benefits of BEMER that persist over time. Second, the study does not provide data on how exposure to disease-modifying therapy (DMT) may impact sexual dysfunction in patients with MS. Specifically, DMT could influence disease progression and symptom severity, potentially improving sexual function [65]. While the sample size (n = 52) meets statistical requirements, its relatively small size may limit the generalizability of the findings. To validate these results and explore long-term effects, larger, multi-center studies are needed. The three-week intervention period is also relatively short, and the lack of long-term follow-up prevents us from assessing the sustainability of the therapeutic effects. Given the fluctuating nature of ED in MS patients, longer-term studies are essential. Another limitation is the absence of additional study groups, such as a group without pelvic floor exercises or one using only BEMER therapy, which would have provided a clearer understanding of each component’s individual contribution to the outcomes.

## 11. Conclusions

This study provides evidence that BEMER therapy can be used as a treatment option for men with MS who have ED, as demonstrated by improvements in all the outcome measures. There appears to be a communication gap between healthcare professionals and patients with MS when it comes to discussing ED. Future studies should investigate the long-term effects of BEMER on a large and diverse population and explore the underlying mechanism of the therapy.

## Figures and Tables

**Table 1:** The baseline characteristics of the study population at randomization.

Characteristic	BEMER (n = 22)	Sham BEMER (n = 23)	*p*-Value
Age (years) *	32.14 ± 4.9	31.91 ± 5.2	0.882
BMI (kg/m^2^) *	26.6 ± 4.6	22.1 ± 2.3	0.001
Disease duration (years) *	6.27 ± 3.7	7.91 ± 4.81	0.211
EDSS *	1.95 ± 1.43	2.57 ± 1.37	0.152
Smoking			0.428
Yes	8 (36.36%)	10 (43.48%)	
No	14 (63.64%)	13 (56.52%)	
Exercise			0.421
Yes	7 (31.82%)	9 (39.13%)	
No	15 (68.18%)	14 (60.87%)	
Classification of ED based on SHIM			
Mild-to-Moderate ED	15 (68.18%)	15 (65.22%)	
Moderate-to-Severe ED	7 (31.82%)	8 (34.78%)	
Classification of SD based on MSISQ-19.			
Primary SD	1 (4.55%)	4 (17.39%)	
Secondary SD	8 (36.36%)	16 (69.57%)	
Tertiary SD	13 (59.09%)	3 (13.04%)	
Did a doctor ask if a patient has symptoms of SD or ED?			0.489
Yes	1 (4.55%)	0 (0%)	
No	21 (95.45%)	23 (100%)	
Did a patient inform their physician about their ED?			0.252
Yes	3 (13.64%)	6 (26.09%)	
No	19 (86.36%)	17 (73.91%)	
Did a patient prefer the gender of a physician when discussing or treating ED?			
Male	15 (68.18%)	16 (69.57%)	
Female	1 (4.55%)	0 (0%)	
There is no difference	6 (27.27%)	7 (30.43%)	

BEMER: bio-electromagnetic energy regulation; BMI: body mass index; EDSS: expanded disability status scale; Exercise: an individual performing more than 150 min of exercise 3 times a week; ED: erectile dysfunction; SHIM: sexual health inventory for men; SD: sexual dysfunction; MSISQ-19: Multiple Sclerosis, Intimacy, and Sexuality Questionnaire. * Data are presented as frequency (%) except for age, BMI, disease duration, and EDSS (mean ± standard deviation).

**Table 2:** Comparison of International Index of Erectile Function—Erectile Function; Sexual Health Inventory for Men; Erection Hardness Scale; The Arizona Sexual Experience Scale; The Multiple Sclerosis, Intimacy, and Sexuality Questionnaire; and Modified Fatigue Impact Scale between groups.

Variables	Group	Change from BAseline Mean (95% CI)	Difference in Mean Change (95% CI)	*p*-Value	Partial Eta Squared
IIEF-EF	BEMER	−7.5 (−9.8, −5.1)	−6.9 (−10.2, −3.7)	<0.001	0.310
	Sham	−0.5 (−2.8, 1.8)			
SHIM	BEMER	−6.6 (−8.6, −4.5)	−6.1 (−8.9, −3.3)	<0.001	0.317
	Sham	−0.5 (−2.4, 1.5)			
EHS	BEMER	−0.9 (−1.2, −0.7)	−0.4 (−0.8, −0.0)	0.022	0.122
	Sham	−0.5 (−0.8, −0.2)			
AXES	BEMER	4.7 (2.9, 6.5)	2.9 (0.3, 5.4)	0.030	0.110
	Sham	1.8 (0.0, 3.6)			
MSISQ-19	BEMER	14.7 (9.1, 20.3)	15.0 (7.0, 22.9)	<0.001	0.261
	Sham	−0.3 (−5.9, 5.3)			
MFIS	BEMER	24.2 (16.5, 31.8)	31.0 (20.2, 41.9)	<0.001	0.456
	Sham	−6.9 (−14.5, 0.8)			

CI: confidence interval; IIEF-EF: international index of erectile function—erectile function; BEMER: bio-electromagnetic energy regulation; SHIM: Sexual Health Inventory For Men; EHS: Erection Hardness Scale; AXES: Arizona Sexual Experience Scale; MSISQ-19: Multiple Sclerosis, Intimacy, and Sexuality Questionnaire; MFIS: Modified Fatigue Impact Scale.

## Data Availability

Data are available upon reasonable request.

## Abbreviations

Abbreviations	Definition
ANCOVA	Analysis of covariance
AXES	Arizona Sexual Experience Scale
BEMER	Bio-electromagnetic energy regulation
BMI	Body mass index
CNS	Central nervous system
CONSORT	Consolidated Standards of Reporting Trials
ED	Erectile dysfunction
EDSS	Expanded Disability Status Scale
EHS	Erection Hardness Scale
IIEF-EF	International Index of Erectile Function—Erectile Function
IRB	Institutional Review Board
MD	Mean difference
MFIS	Modified Fatigue Impact Scale
MS	Multiple sclerosis
MSISQ-19	Multiple Sclerosis, Intimacy, and Sexuality Questionnaire
SD	Sexual dysfunction
SHIM	Sexual Health Inventory for Men
SNOSE	Sequentially numbered concealed opaque envelope
